# Annual Variability of Wing Morphology in* Culex sitiens* Wiedemann (Diptera, Culicidae) Mosquito Vectors from the Coastal Area of Samut Songkhram Province, Thailand

**DOI:** 10.1155/2019/3978965

**Published:** 2019-03-03

**Authors:** Tanawat Chaiphongpachara, Sedthapong Laojun

**Affiliations:** ^1^College of Allied Health Science, Suan Sunandha Rajabhat University, Samut Songkhram 75000, Thailand; ^2^Bachelor of Public Health, College of Allied Health Sciences, Suan Sunandha Rajabhat University, Samut Songkhram 75000, Thailand

## Abstract

*Culex sitiens* Wiedemann (Diptera, Culicidae) is a mosquito vector that is found in coastal areas. Effective control of mosquitoes requires knowledge of the biology, ecology, and behavior of the vector as well as of various other aspects, including its morphology. Currently, variations in the wing size and shape of coastal* Cx. sitiens* have not been described. Here, morphological changes were studied in the wings of* Cx. sitiens* from a coastal area of Samut Songkhram Province, Thailand. Samples were collected at night (6:00 pm–6:00 am) during single weeks of September in the years 2015–2017 using Center for Disease Control light traps with dry ice as bait. Eighteen landmarks of each individual were selected and digitized for landmark-based geometric morphometric analyses. Wing size variability was estimated using the isometric estimator of centroid size. Wing-shape variables were computed as Procrustes superimposition with residual coordinates of the 18 landmarks following a Generalized Procrustes Analysis and the principal components of residual coordinates. Degrees of wing-shape dissimilarity among individuals were analyzed using discriminant analysis or canonical variate analysis, which was illustrated in a discriminant space of canonical variables. Differences in wing size and shape among populations were calculated using nonparametric permutations based on 1000 runs with Bonferroni correction tests at a* p*-value of <0.05. The wing sizes and shapes of the mosquitoes differed significantly between observation years in all population groups, as indicated by nonparametric tests (1000 runs) with the Bonferroni correction. Differing rainfall between observation years was related to morphological changes in mosquito populations, presumably reflecting environmental adaptation. Differences in the wing morphology of* Cx. sitiens* between annual populations reflect adaptation to environmental variables such as rainfall and may affect the potential to act as insect vectors of human disease. These observations may facilitate the development of tools for managing mosquito-borne disease.

## 1. Introduction

*Culex sitiens* Wiedemann (Diptera, Culicidae) is a mosquito vector that is distributed throughout coastal areas in countries including Thailand, India, Indonesia, Malaysia, Vietnam, Myanmar, Cambodia, Australia, Papua New Guinea, and New Zealand [1–3].* Cx. sitiens* is a prominent coastal vector of many mosquito-borne diseases [1], including Japanese encephalitis (JE) [4]. As described previously, primary vectors of JE include* Cx. fuscocephala*,* Cx. tritaeniorhynchus*,* Cx. gelidus*, and* Cx. vishnui* and generally breed in rice fields [5]. In 1994,* Cx. sitiens* brackish water mosquitoes were, for the first time, found positive for the JE virus in Sabak Bernam, Selangor, Malaysia [6]. In addition, laboratory studies showed that* Cx. sitiens* can transmit the JE virus during its blood feeding. In contrast,* Cx. sitiens *showed no potential to act as a vector for filariasis, although a filarial nematode infection was reportedly transmitted by* Cx. sitiens* in Thailand, albeit with signs of degeneration in the parasite larvae [7]. Consistent with this, Prummongkol* et al*. [8] showed that* Cx. sitiens* are resistant to experimental infection (0% infected) by lymphatic filariasis. In 2014, the World Health Organization [9] reported that more than 3 billion people have been exposed to risks of Japanese encephalitis infection.

Effective control of mosquitoes requires a variety of entomological knowledge, including the biology, ecology, and behavior of this vector [13]. Studies of relationships between environmental changes and morphological changes in mosquitoes are crucial to the understanding of their behaviors as vectors [14]. However, it remains unclear whether* Cx. sitiens* varies in size or shape depending on the environmental conditions in coastal areas.

Samut Songkhram Province is in central coastal Thailand on the Gulf of Thailand. This area is a suitable habitat for the brackish water mosquito* Cx. sitiens, *which causes various medical problems in the area [1]. Abnormal weather conditions are increasingly observed in many countries, and the associated changes in temperature, humidity, and rainfall have been attributed to global warming [15]. Abnormal yearly fluctuations in weather conditions may affect the ability of coastal mosquitoes to act as vectors and may demand greater adaptation for survival. This research contributes to the understanding of the morphological variability of* Cx. sitiens* and increases the knowledge of this species as a coastal vector of human diseases.

## 2. Materials and Methods

### 2.1. Mosquito Collection and Identification

**Figure 1:**
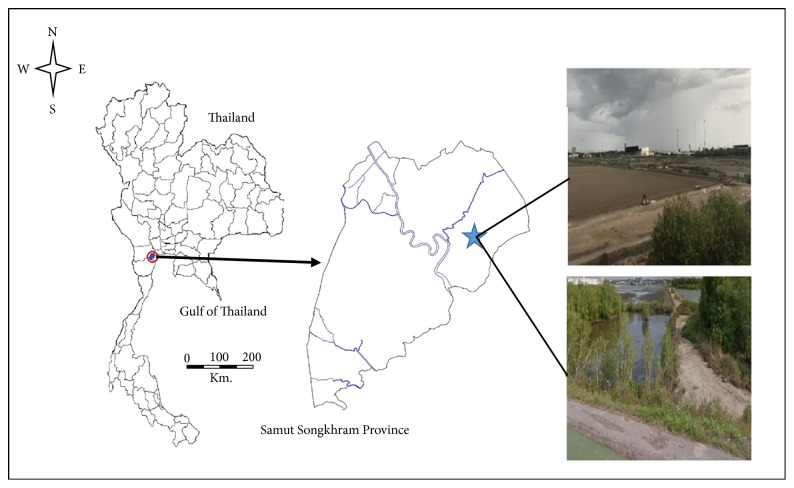
Study sites and sampling locations in Samut Songkhram Province.

Mosquitoes were collected from the coastal community area of Samut Songkhram Province, Thailand (13°24′34.3′′N 100°00′52.9′′E; Figure 1). Samples were collected during single weeks of September in the years 2015–2017 at night (6:00 pm–6:00 am), using Center for Disease Control (John W. Hock co., FL) traps with dry ice as bait. The range of annual rainfall for Samut Songkhram Province was 800–1000 mm [10], 1000–1400 mm [11], and 1200–1600 mm [12] for 2015–2017, respectively, as shown in Table 1 along with average annual temperature ranges. Two traps were hung 50 m from randomly selected houses at a height of 1.5 m. Mosquitoes were collected in the morning and were sent to the laboratory at the College of Allied Health Sciences, Suan Sunandha Rajabhat University, Samut Songkhram Provincial Education Center after recording the total numbers of mosquitoes in the traps.* Cx. sitiens* mosquitoes were then identified based on morphology using the Illustrated Keys [16] and a Nikon AZ 100 M stereomicroscope (Nikon Corp., Tokyo, Japan). Only female* Cx. sitiens* were used for geometric morphometric (GM) analyses in this study. A total of 116 female* Cx. sitiens *specimens were analyzed, including 26 mosquitoes from 2015, 26 mosquitoes from 2016, and 64 mosquitoes from 2017.

### 2.2. Sample Preparation for Landmark-Based GM Analyses

In this study, to avoid interference in the analysis from intraindividual variation, only right wings were used because in our samples, left wings were more damaged than the right wings. The right wings of* Cx. sitiens *were removed from the thorax and mounted using Hoyer's medium in the center of microscope slides to avoid peripheral optical distortion. The wings were then photographed using a digital camera connected to a Nikon SMZ745T stereomicroscope (Nikon Corp., Tokyo, Japan) at 40× magnification. A scale bar (1 mm in length) was added to the wing images and used to convert the coordinates from pixels to millimeters.

### 2.3. Landmark Digitizing and Repeatability Tests

Eighteen landmarks on each individual following Demari-Silva et al. [17] were selected and digitized (Figure 3(a)). After digitizing the landmarks of all wings, 20 wing samples were randomly selected from each year and landmark-digitized a second time. The repeatability of the digitizing procedure was assessed by comparing the digitization datasets for these twice-digitized samples using the repeatability (R) index, which is a Model II one-way ANOVA for repeated measures [18, 19].

### 2.4. Wing Size Analyses

Wing size variability was estimated using the isometric estimator of centroid size (CS), which is defined as the square root of the sum of the squared distances from the centroid to each landmark [14, 20]. The CS variations of* Cx. sitiens *populations in each year are presented in boxplots. CS differences between sampling years were identified in three pairwise comparisons using nonparametric tests based on 1000 runs with Bonferroni correction tests at a* p*-value of < 0.05. In addition, CS of samples has been analyzed to inspect the relationship with annual rainfall and annual average temperature using Pearson's correlation coefficient at a* p*-value of < 0.05.

### 2.5. Wing-Shape Analyses

Wing-shape variables (also called tangent space variables or Procrustes residuals) were computed as Procrustes superimpositions with the residual coordinates of the 18 landmarks following a Generalized Procrustes Analysis and the principal components of the residual coordinates (after their orthogonal projection onto the Euclidean plane tangent to the consensus form). Degrees of wing-shape dissimilarity among individuals were analyzed using discriminant analysis or canonical variate analysis, which was illustrated in a discriminant space of canonical variables. Mahalanobis distance scores were computed from the discriminant analysis, which was used to estimate shape divergences. Differences in Mahalanobis distances among populations were calculated using nonparametric permutations based on 1000 runs with Bonferroni correction tests at a* p*-value of < 0.05, while mean shape variables of samples have been analyzed to examine the relationship with annual rainfall and annual average temperature using Pearson's correlation coefficient at a* p*-value of < 0.05.

A cross-validated classification (jackknife classification) was used to test the accuracy of group classifications from geometric morphometrics [21]. For this procedure, individuals were reclassified according to their closest groups based on Mahalanobis distances without being used to help determine a group center. To illustrate morphological divergence among* Cx. sitiens* populations between study years, a single-linkage hierarchical classification tree was built, the significance of which was tested by the bootstrap as described by Couette et al. [22]. Twenty* Cx*.* quinquefasciatus* wings from the Ratchaburi Province were used for the outgroup.

The allometric effect in this study was explored by regression of the Procrustes components factors on centroid size and computing the coefficient of determination.

### 2.6. Software

Landmark-based GM analyses were performed using the recent online morphometric package XYOM, which is freely available at https://xyom.io/me. The MOG module in the CLIC package available at https://xyom-clic.eu was used to test for allometry. Pearson's correlation coefficient was used to examine the relationship between morphology (size and shape) and climate (rainfall and temperature) using SPSS version 17 (SPSS Inc., Chicago).

## 3. Results

A total of 116* Cx. sitiens* specimens were analyzed. Measurement errors were less than 5% in CS estimations (for the years 2015, 2016, and 2017, with repeatability indices of 0.96, 0.93, and 0.93, respectively) and were less than 10% in shape (relative warps) estimations (2015, 2016, and 2017, with repeatability indices of 0.93, 0.91, and 0.90, respectively).

### 3.1. Size Variability

**Figure 2:**
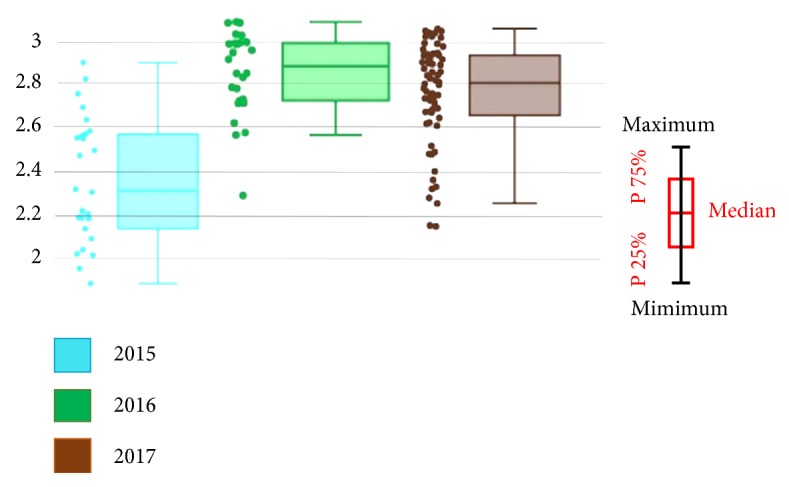
Boxplots representing wing CS variations in each study year; the boxplots show the median scores and 25^th^ and 75^th^ quartiles.

The wing CS varied between* Cx. sitiens* populations from each year (Figure 2). Specifically, the 2016* Cx. sitiens *population had the highest average CS (2.89 mm), followed by 2017 (2.78 mm) and then 2015 (2.35 mm; Table 2). The wing CS of* Cx. sitiens* differed significantly between all study years (*p* < 0.05). The statistical relationship between CS and climate data revealed that the annual rainfall was related to the CS (*p* < 0.01,* r *= .488).

### 3.2. Shape Variability

**Figure 3:**
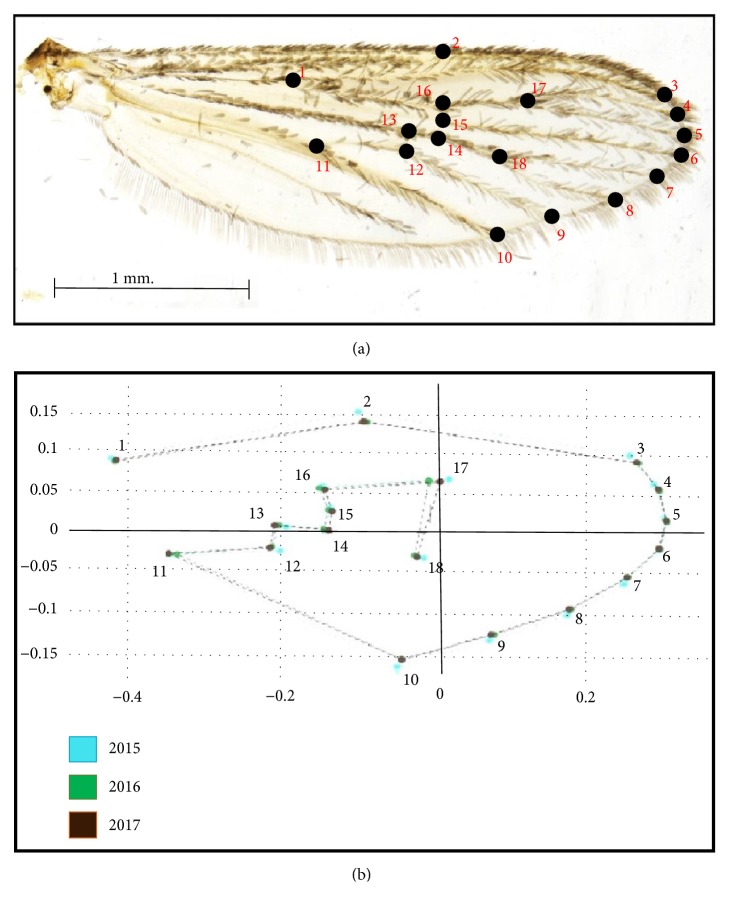
(a) The positions of 18 landmarks on the wing of* Cx. sitiens* used for geometric morphometric analyses. (b) Configurations of those 18 landmarks connected by a straight line after Procrustes superimposition of the* Cx. sitiens* population in each year.

**Figure 4:**
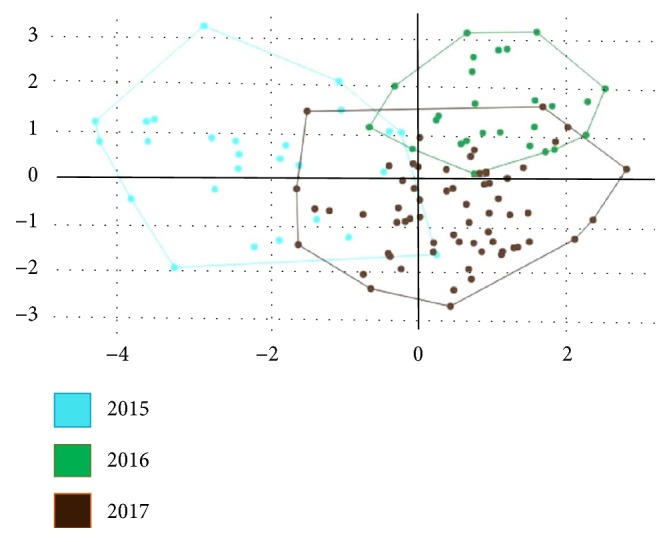
Discriminant space of canonical variates by DA of wing principal components resulting from comparison of the variation in shape in* Cx. sitiens* populations from each year.

**Figure 5:**
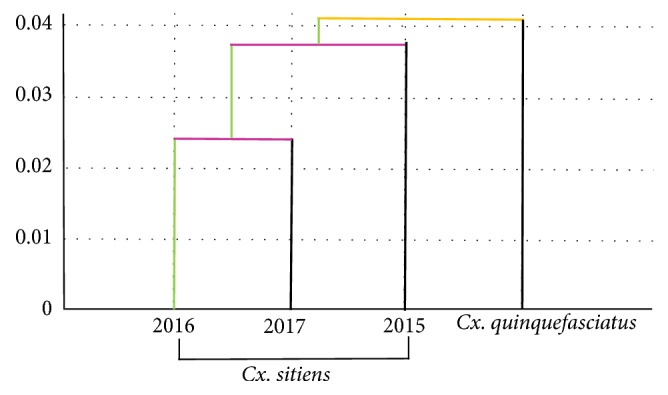
Single-linkage hierarchical classification tree of* Cx. sitiens* wing shapes in each study year. The wing shapes of* Cx. quinquefasciatus *wings from the Ratchaburi Province are included as an outgroup.

In Figure 3(b), we present the mean annual configurations of the 18* Cx. sitiens* wing landmarks with connecting lines for the Procrustes analyses. Comparisons of these landmarks and their connecting lines indicate that among the 18 wing landmark displacements (Figure 3(b)). Subsequent discriminant analyses revealed differing shape differentiation of* Cx. sitiens* wings between study years (Figure 4). Moreover, the Mahalanobis distance scores of the wing shapes in each year differed significantly in all pairwise comparisons (*p* < 0.01; Table 3). Accordingly, cross-validated classification scores of the Mahalanobis distances ranged from 61% to 69% and were higher in the years 2016 (69%; Table 4). Finally, the single-linkage hierarchical classification tree based on Euclidean distances between groups separated* Cx. sitiens* populations in 2015 from populations in 2016 and 2017 (Figure 5*).*

**Figure 6:**
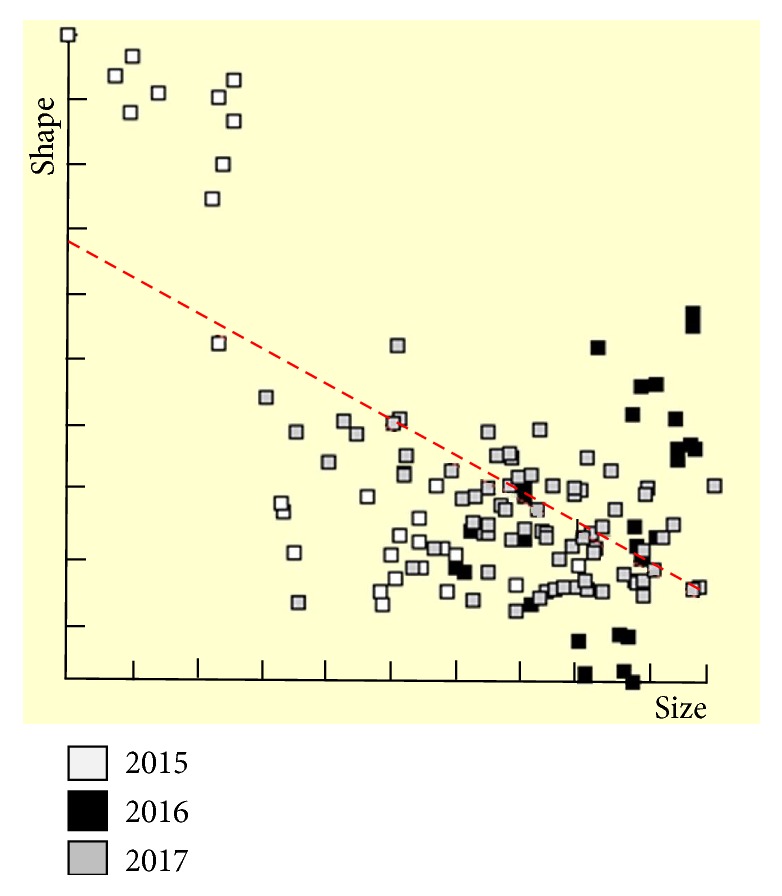
An allometric plot. Each point represents an individual* Cx. sitiens *sample, symbolized by collection year. The horizontal axis is the CS; the vertical axis is the first PC (principal component of Procrustes residuals).

The allometric residual of the first discriminant factor was 41% (t = -8.98, df = 114, p = 3.22), which is the effect of size variation on shape. An allometric plot from the calculation of allometry is shown in Figure 6, while Pearson's correlation coefficient revealed statistical relationship between shape and annual rainfall (*p *< 0.05,* r* = .201).

## 4. Discussion

We collected individuals of* Cx. sitiens*, a JE vector, from coastal populations during the Septembers of 2015, 2016, and 2017 and studied morphological differences between the study years. The GM results of this study reveal clear differences in morphology, such as size and shape, in each year.

*Cx. sitiens* wing CS differed significantly between population groups, as indicated by nonparametric tests (1000 runs) with the Bonferroni correction (*p* < 0.05). Our study has found a relationship between morphology (both size and shape) and rainfall* (p* < 0.05). Coastal habitats are strongly influenced by weather and other seasonal environmental factors throughout the year [1]. Environmental factors have previously been shown to directly affect mosquito body sizes, especially those at breeding grounds during the larval stage [23, 24], such as larval habitat quality and larval competition. During the study years, the weather conditions at the Samut Songkhram provincial meteorological station were distinguished by differing rainfall totals. Because* Cx. sitiens* predominantly inhabits stagnant salt ponds or water sources near mangrove forests, which are abundant during the rainy season [1], differing rainfall totals between the study years may have influenced our morphological observations. Previously, differences in the wing CS of* Anopheles coluzzii* populations between rainy and dry seasons were reported in Burkina Faso (West Africa) [25]. In this study,* Cx. sitiens* populations from 2015 had the smallest wings as well as the lowest range of annual rainfall of Samut Songkhram Province. Rainfall is an important factor that is associated with abundant breeding sites for mosquito vectors and ensures a suitable relative humidity [26]. In 2015, the annual rainfall was less than the normal criteria by 50–100 mm (shown in Table 1). Our observations are consistent with previous research on various factors affecting the body size of* Aedes albifasciatus* in Central Argentina, which found that body size variation depends on the aquatic habitat where immature stages develop, influenced more by rainfall than by other environmental factors [27]. Other work has also identified temperature as a factor related to the wing size and shape of mosquitoes [22]. However, in this work, during the years 2015–2017, the range of average temperatures in each year did not differ among years (28–30°C). Our results also demonstrate significant impacts of environmental factors, especially rainfall, on size variations of a mosquito vector. The body size of mosquito vectors is important in the medical entomology context because it is associated with the mosquito's longevity, fecundity, and the size of its blood meals. Additionally, smaller mosquitoes may have more limited abilities to transmit human pathogens [28].

Wing shapes differed across all pairs of the mosquito populations, and comparisons of Mahalanobis distances (*p*-value < 0.01) corroborated these differences, suggesting environmental influences, especially rainfall. With the progression of global warming, greater differences in weather patterns are expected from year to year, warranting further studies of adaptive mechanisms, including those of mosquitoes [12]. In a study of wing-shape diversity in* Cx. coronator* from south and southeast Brazil, Demari-Silva et al. [17] suggested that variations between the study areas reflect climate differences. Our morphological tree analyses indicate similar wing shapes of* Cx. sitiens* mosquitoes in 2016 and 2017, but differing wing shapes in 2015. In agreement with previous studies showing environmental impacts on insect morphology, the 2015 annual rainfall was 50–100 mm less than the normal criteria in Thailand. Gomez et al. [29] similarly reported that rainfall influences the wing shape of the Columbian malaria vector* Anopheles albimanus*. Taken together, these observations of environmentally driven morphological differences in potential disease vectors warrant further studies of the environmental adaptations of mosquitoes.

## 5. Conclusions

Herein, we demonstrate morphological variability in* Cx. sitiens* wings collected in the years 2015–2017 in the coastal area of Samut Songkhram Province, Thailand. Differences in wing size and shape corresponded to differences in weather conditions, especially rainfall, among the study years, and likely reflect adaptation to the environment. Although we did not perform genetic or epigenetic studies, these changes in the size and shape of* Cx. sitiens* wings likely correspond with the effects of rainfall on habitat availability. However, further studies of morphological differences are required to compare mosquitoes from differing environments and years using different sample types over time. These studies may confirm the effects of weather conditions on mosquito vector morphology.

## Figures and Tables

**Table 1:** Annual rainfall and annual average temperature ranges of Samut Songkhram Province in 2015–2017.

Year	Annual rainfall range	Comparison with the normal criteria of Thailand	Annual average temperature range	Comparison with the normal criteria of Thailand
2015	800–1000 mm	50–100 mm less than normal criteria [10]	28–30°C	0.5°C higher than normal criteria [10]
2016	1000–1400 mm	100–200 mm more than normal criteria [11]	28–30°C	0.5°C higher than normal criteria [11]
2017	1200–1600 mm	200–400 mm more than normal criteria [12]	28–30°C	0.5°C higher than normal criteria [12]

According to the data of the Meteorological Department of Thailand, the normal criteria for annual rainfall and annual average temperature are the thirty-year averages (1981–2010). ^†^mm, millimeter; ^‡°^C, degrees Celsius.

**Table 2:** Mean differences in the wing CS among *Cx. sitiens *populations from each study year.

Years	Mean ± SD (mm)	min–max (mm)
2015	2.35 ± 0.08^a^	1.87–2.89
2016	2.89 ± 0.02^b^	2.45–3.06
2017	2.78 ± 0.05^c^	2.22–3.04

Different superscript letters are statistical differences at *p* < 0.05 (*p* < 0.05); ^†^SD, standard deviation; ^‡^min, minimum; ^§^max, maximum.

**Table 3:** Mahalanobis distances for differences in wing shapes of *Cx. sitiens* between study years.

Years	2015	2016	2017
2015	-		
2016	3.60*∗*	-	
2017	3.04*∗*	2.50*∗*	-

*∗* = significant differences at *p *< 0.05.

**Table 4:** Cross-validated reclassification scores based on wing shape similarities in *Cx. sitiens* populations from the three study years.

Years	Assigned	Observed	Classification Accuracy (%)
2015	16	26	61
2016	18	26	69
2017	42	64	65

## Data Availability

The data supporting the conclusions of this article are provided within the article. The datasets generated and analyzed during the current study are available from the corresponding author upon reasonable request.
